# Ferroptosis regulator NOS2 is closely associated with the prognosis and cell malignant behaviors of hepatoblastoma: a bioinformatic and *in vitro* study

**DOI:** 10.3389/fonc.2023.1228199

**Published:** 2023-09-19

**Authors:** Lan Zhang, Bin-cheng Ren, Fei Wei, Yan Liu, Ya Gao, Bo Yuan

**Affiliations:** ^1^Department of Pediatrics, Second Affiliated Hospital of Xi’an Jiaotong University, Xi’an, China; ^2^Department of Rheumatology and Immunology, Second Affiliated Hospital of Xi’an Jiaotong University, Xi’an, China; ^3^Department of Pediatric Surgery, The Second Affiliated Hospital of Xi’an Jiaotong University, Xi’an, Shaanxi, China; ^4^Department of General Surgery, Xi’an Central Hospital, Xi’an, China

**Keywords:** hepatoblastoma, ferroptosis, NOS2, clinical assessment, malignant progression

## Abstract

**Background:**

Hepatoblastoma (HB) is the most common liver tumor in children with easy metastasis. The emergence of ferroptosis as a novel form of cell death has gained increased attention in various human cancers. However, the roles of ferroptosis-related (FR) genes in HB remain elusive

**Methods:**

The GSE133039, GSE131329, and GSE81928 datasets were utilized for screening core FR genes in HB. Through Lasso regression analysis and using the support vector machine recursive feature elimination (SVM-RFE) algorithm, three candidate FR genes were obtained for characterizing HB. Their expression patterns and their clinical associations were explored through the ‘Limma’ R package, and their diagnostic potential was evaluated using ROC curves. Nitric oxide synthase 2 (NOS2) emerged as a candidate for further analyses. The CIBERSORT algorithm and GSEA dataset were used to respectively investigate the immune and metabolism effects of NOS2; the former was validated through immunofluorescence. The GSDC database was employed to analyze the correlation between NOS2 expression and the therapeutic efficacy of multiple drugs. PCR, Western blotting, colony formation assays, and Transwell experiments, were used to determine biological functions of NOS2 in HB cells. Potential upstream transcription factors of NOS2 were predicted through the TRRUST, hTFtarget, GeneCards, and JASPAR databases.

**Results:**

NQO1, SLC1A4, and NOS2 were identified as potential genes in HB and found to be significantly upregulated in tumor samples. Nevertheless, only NOS2 was closely associated with HB clinicopathological characteristics; high NOS2 expression indicated poor prognosis, metastatic tendency, and late clinical stage. Immune analyses indicated that high NOS2 expression was concomitant with decreased infiltration levels of CD8+ T cells but increased infiltration levels of macrophages. GSEA revealed that NOS2 failed to affect the enrichments of glycolysis, fatty acid metabolism, and cholesterol biosynthesis in HB. Moreover, NOS2 was positively correlated with the IC_50_ values of trametinib, lapatinib, and cisplatin. NOS2 overexpression promoted the proliferation, migration and invasion of HepG2 and HuH-6 cells. JUND was identified as a potential transcriptional regulator of NOS2 by binding to its promoter (5’-TTCTGACTCTTTT-3’).

**Conclusion:**

NOS2 plays a significant role in HB clinical assessments and holds promise as a novel therapeutic target.

## 1 Introduction

Hepatoblastoma (HB) is the most common pediatric liver tumor with an incidence of 1.2 per million (1). Most HB cases are diagnosed under the age of three years, with dominant clinical features including feeding intolerance, emesis, and the presence of abdominal mass (2). The primary approach for treating HB involves a combination of surgical resection and chemotherapy. This strategy is effectively supported by the use of cisplatin, doxorubicin, vincristine, irinotecan, and 5-fluorouracil to form the mainstay of HB treatments (3). With continuous improvements in therapeutic approaches, the overall survival (OS) rate of children with HB has increased up to 80% (4). Nevertheless, due to the high heterogeneity and malignancy, one-third of children with HB will suffer from metastatic disease (3). Furthermore, up to half of these advanced cases present with pulmonary metastases (5). Despite receiving curative resection, the 5-year OS rate of these children is still less than 56% (6). Therefore, it is imperative to identify effective biomarkers for HB clinical assessments and to develop novel therapeutic targets.

Ferroptosis is a novel form of programmed cell death characterized by distinct mitochondrial microstructural variations, such as reduced mitochondrial ridges and increased mitochondrial density (7). This process is mediated by three fundamental components: iron dependence, lipid peroxidation, and antioxidant pathway imbalance, acting as the core links of ferroptosis (8). As research into this phenomenon deepens, considerable evidence has emerged highlighting its multifaceted roles in various cancer types.

In hepatocellular carcinoma (HCC), for instance, SOCS2 increases radiotherapy sensitivity by enhancing SLC7A11 ubiquitination and ferroptosis (9). The ferroptosis regulatory gene SLC1A5 promotes the malignant progression of pancreatic adenocarcinoma through activation of the mTORC1 signaling pathway (10). Moreover, a newly established ferroptosis score could predict chemotherapy and anti-tumor immune responsiveness in colorectal cancer (11).

Regrettably, extremely limited research has explored the links between ferroptosis and HB. To our knowledge, only two studies have focused on this scope. One study revealed that N6-methyladenosine modification enhances HB resistance to ferroptosis by inhibiting SLC7A11, thereby mediating therapeutic resistance (12). Another bioinformatic study reported that the ferroptosis driver ATF3 may serve as a biomarker for predicting HB occurrence (13).

Given the above context, this study aimed to investigate the roles of core ferroptosis-related (FR) genes in HB clinical assessment and malignant progression. Through a series of data mining and cell experiments, our research identified nitric oxide synthase 2 (NOS2) as a promising biomarker for diagnosing and evaluating HB. NOS2 was closely associated with disease status, immune response, and treatment outcomes. Additionally, its role in stimulating HB progression was confirmed, thereby showing a novel avenue for targeted interventions. Our findings undoubtedly contribute valuable insights into HB assessment and treatment strategies.

## 2 Materials and methods

### 2.1 Data source

Three GEO datasets, including GSE133039, GSE131329, and GSE81928, were applied for data mining. A brief description of each and their uses are shown in **Supplementary Table 1**. The clinical characteristics of these datasets are presented in **Supplementary Tables 2**-**3**. All transcriptome data was standardized by log2 (FPKM+1) transformation.

### 2.2 FR gene set

An FR gene set was constructed utilizing 253 homo-sapiens ferroptosis genes from FerrDb (http://www.zhounan.org/ferrdb/current/), recognized as the most comprehensive database dedicated to ferroptosis regulators and ferroptosis-disease associations (14). The protein-protein interaction (PPI) network of these FR genes was depicted through the STRING database (https://string-db.org/) (15) and Cytoscape v3.71 software (16). Their biological functions were analyzed using the online Metascape tool (http://metascape.org/) (17).

### 2.3 Consensus clustering analysis

Consensus clustering analysis was employed for identifying HB diagnostic patterns based on the expressions of FR genes. This analysis was accomplished by using the ‘ConsensusClusterPlus’ package in R v4.1.2 (18). The optimal number of clusters could be determined according to the K-means algorithm and cumulative distribution function (CDF) (18). A heatmap of the consistency matrix was applied to visualise the intra- and intergroup variabilities within each cluster.

### 2.4 Screening core genes in HB

In the present study, two approaches were combined to identify the core FR genes for characterizing HB, including Lasso regression analysis and the support vector machine recursive feature elimination (SVM-RFE) algorithm. The Lasso regression analysis was conducted using the ‘glmnet’ R package based on the 7-fold cross-validation scheme. SVM-RFE, a common machine-learning method, was used to reduce the number of parameters by creating weight vectors for each parameter. These weights determine the importance of the parameters, thereby maximizing data separation via an arithmetical hyperplane (19). SVM-RFE was performed using the ‘KeBABS’ R package (20).

### 2.5 Clinical correlation analyses

Considering that only the GSE131329 dataset harbored HB survival outcomes, yet without survival time, the differences in expression of three candidate genes between deceased and surviving cases were compared for this dataset. Their predicted accuracy was also assessed using the receiver operating characteristic (ROC) curve. The relationships between the expression of the three core genes and HB clinicopathological features were analyzed based on the analysis of variance test, including histological type, metastatic event, and clinical stage.

### 2.6 Immune bioinformatic analysis and immunofluorescence assay

The changes in infiltration levels of 21 immune cells were evaluated through the CIBERSORT algorithm, which quantifies the cell composition of sample tissue based on the gene expression profiles (21).

To verify the immunogenic effects of NOS2, we performed immunofluorescence detections on two pairs of clinical samples with different NOS2 protein levels. Immunofluorescence was conducted as previously described (22). Briefly, 4-mm tissue sections of HB clinical samples were prepared, and dewaxed with xylene and anhydrous ethanol. Then, the tissue sections underwent antigen repair, fixation, and nonspecific blocking. Next, the sections were incubated with first and secondary antibodies (NOS2, ab178945; CD8, ab33786; CD163, ab87099; Abcam, Cambridge, UK) in conditions protected from light. The slides were then sealed with anti-fluorescence quenching sealing tablets. CD8 and CD163 were stained with the Cy3-labeled goat anti-mice IgG (H&L) antibody (P0193; Beyotime, Shanghai, China). NOS2 was stained with the FITC-labeled goat anti-rabbit IgG (H&L) antibody (P0186; Beyotime). Nuclei were stained with Hoechst reagent (C0003; Beyotime). Subsequently, the slides were analyzed with an automatic fluorescent microscope using a 40× objective lens (BX53; Olympus, Tokyo, Japan).

### 2.7 Gene set enrichment analysis

GSEA was used to probe into the effects of NOS2 on multiple metabolic processes, including glycolysis, fatty acid metabolism, and cholesterol biosynthesis. The corresponding gene sets were obtained from the MsigDB database (https://www.gsea-msigdb.org/) and their detailed descriptions are presented in **Supplementary Table 4**. The parameters for GSEA analyses were set as follows: phenotype labels set as high- versus low-NOS2 expression samples; the number of permutations 1000; and no collapse in gene symbols. Transcriptome data were derived from the GSE133039 dataset.

### 2.8 Therapeutic correlation and mutation analyses

The Genomics of Drug Sensitivity in Cancer (GDSC) database (www.cancerRxgene.org) was utilized for the connections between NOS2 and the sensitivity of both molecular targeted therapy (MTT) and chemotherapy drugs (23). Drug sensitivity was based on the half-maximal inhibitory concentration (IC_50_). Correlations were assessed based on Pearson’s coefficient. Mutational features of the three candidate genes in the two pediatric projects (n=1,064 samples) were displayed using the cBioPortal database (http://cbioportal.org) (24).

### 2.9 Regulatory mechanism analysis

The TRRUST database (https://www.grnpedia.org/trrust/) (25), hTFtarget database (http://bioinfo.life.hust.edu.cn/) (26), and a subsection of the GeneCards database termed ‘Promoters/Enhancers’ (https://www.genecards.org/) (27) were employed for predicting the potential upstream transcription factors (TFs) of NOS2. The promoter sequence of NOS2 was obtained from the UCSC database (http://genome.ucsc.edu/). The possible binding sites between the predicted TFs and NOS2 were achieved through the JASPAR database (https://jaspar.genereg.net/) (28). The motif sequence of the candidate TF was also exhibited through the JASPAR database. Potential enhancers of NOS2 were predicted using the EnhancerDB database (http://lcbb.swjtu.edu.cn/EnhancerDB/) (29).

### 2.10 Clinical samples and real-time quantitative polymerase chain reaction (RT-qPCR)

RT-qPCR analyses were conducted on 25 pairs of HB tissues and adjacent normal tissues from the Second Affiliated Hospital of Xi’an Jiaotong University to confirm differential expression of NOS2. Prior to the clinical sample analysis, informed consent was obtained from the parents of the children involved. The study protocol was approved by the Ethics Committees of the Second Affiliated Hospital of Xi’an Jiaotong University. All methods in this study were performed in accordance with relevant guidelines and regulations.

For RNA extraction, TRIzol reagent (Takara, Dalian, China) was employed. The purity of each RNA sample was assessed using the optical density ratio at 260nm and 280nm (A260/A280). Reverse transcription was performed using the PrimeScript RT reagent Kit (Takara). The RT-qPCR reaction was tracked using the SYBR-Green PCR Reagent (Takara) and subsequently detected on an ABI Prism 7900 sequence system. GAPDH was used as the internal reference gene. The relative gene expression was calculated according to the 2^-ΔΔCT^ method. The list of primer sequences is shown in **Supplementary Table 5**.

### 2.11 Western blot

Western blot assays were performed as previously described (10). Transfected cells were lysed using RIPA buffer (Beyotime) while kept on ice. The protein concentration of lysastes was measured using a BCA kit (Phygene Life Sciences Company, Fuzhou, China). Protein samples were separated using 10% SDS-PAGE. After electrophoresis, protein samples were transferred to PVDF membranes (BestBio, Shanghai, China). The PVDF membranes were blocked using 5% skim milk at 37°C for 2 h. The membranes were subsequently incubated with the primary and secondary antibodies, respectively (all purchased from Abcam). The primary antibodies were as follows: rabbit monoclonal anti-NOS2 (1:1,000; ab178945) and rabbit polyclonal anti-GAPDH antibody (1:1,000; ab22555). The secondary antibody was goat-anti rabbit IgG-HRP secondary antibody (1:2,000; ab205718).

### 2.12 Cell culture and transfection

Two HB cell lines (HepG2 and HuH-6) were purchased from Procell Life Company (Wuhan, China). These cells were maintained in distinct culture mediums: HepG2 cells were cultured in MEM medium, while HuH-6 cells were cultured in DMEM medium. Both mediums were supplemented with 10% fetal bovine serum (FBS) and 1% penicillin/streptomycin. The cells were cultured at 37 °C and 5% CO_2_.

For gene manipulation, short hairpin RNA (sh-NOS2) for NOS2 knockdown and overexpression plasmids (OE-NOS2) were designed and synthesized by HanHeng Biotechnology (Shanghai, China). The transfection process followed the manufacturer’s instructions.

### 2.13 Colony formation assay

The colony formation assay was used to evaluate the proliferative abilities of HB cells. Transfected cells in the logarithmic growth phase were seeded into 6-well plates (1×10^3^ cells per well). After 1-week incubation, visible colonies formed. Then, the cells were fixed and stained using methanol and Giemsa, respectively. Colonies were counted under a microscope by examining five random fields.

### 2.14 Transwell migration and invasion assays

The workflow was consistent with that described in previous research (10, 30). For migration assays, mediums containing 0.1% FBS were added to the upper chambers, while medium containing 10% FBS was added to the lower chambers. Cells were seeded in the upper chamber at a density of 1×10^4^ cells per well. After 24 h, non-migrated cells were removed washing with PBS and gently scraping with a cotton swab. The cells adhering to the lower membrane surface represented migrated cells. These cells were fixed by paraformaldehyde for 20 min and stained with 0.1% crystal violet for 20 min. Cell counting was performed under a high magnification microscope (100-fold) from five randomly-selected visual fields.

For invasive assays, the upper chambers were precoated with Matrigel (CORNING, Corning, NY, USA). The subsequent steps were similar to the migration experiment.

## 3 Results

### 3.1 Constructing an FR gene set and screening its core genes

**Figure 1:**
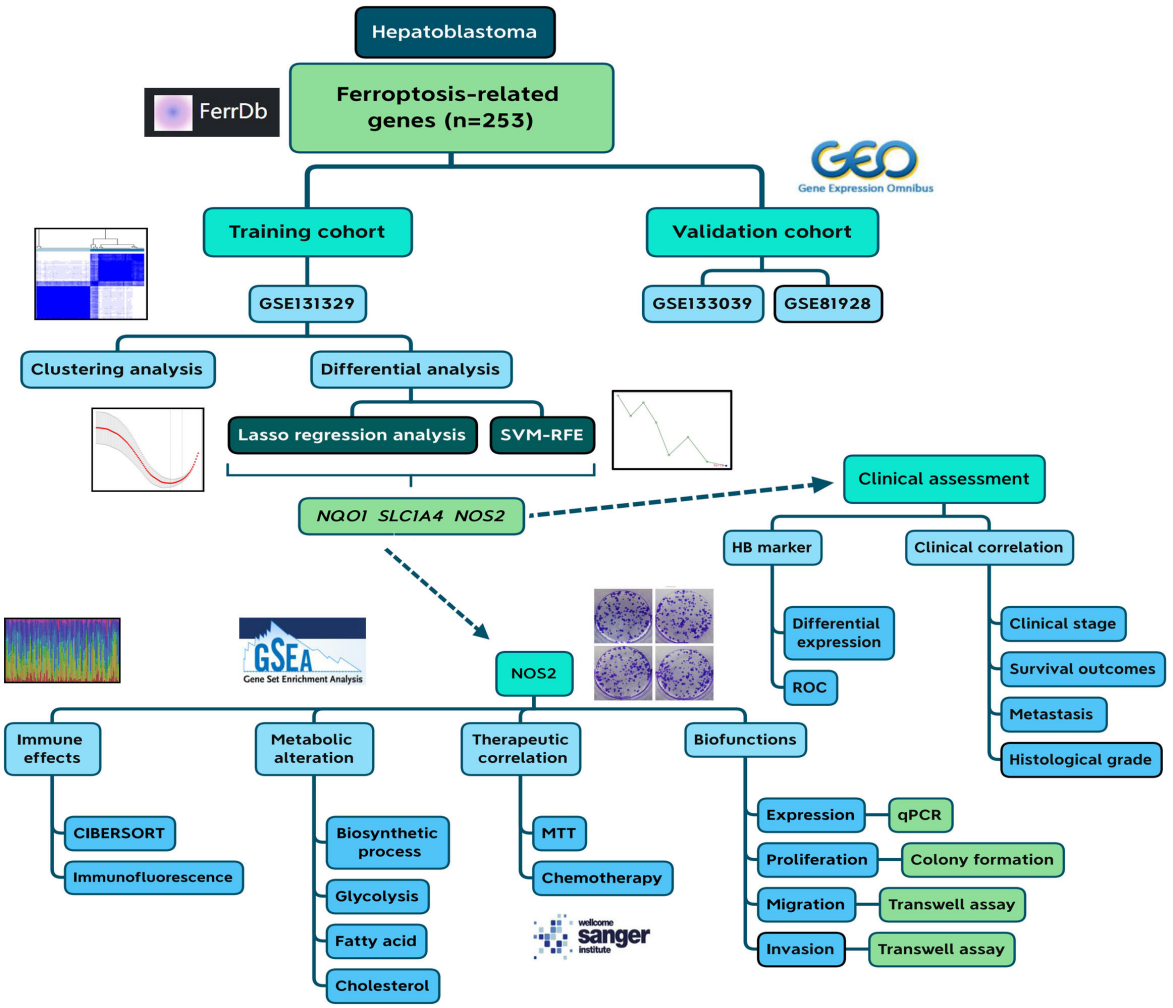
Flow chart of the current study. SVM-RFE, support vector machine recursive feature elimination; HB, hepatoblastoma; ROC, receiver operating characteristic.

The flowchart of this study is presented in **Figure 1**. First, we selected three potential core ferroptosis genes in HB using Lasso regression analysis and the SVM-RFE algorithm based on a comprehensive and reliable FR gene set (n=253). Second, their clinical relevance was explored through three cohorts. Third, NOS2 was selected for further multi-omics investigation, including its impact on immune responses, its associations with HB metabolism, and its role in determining the therapeutic efficacy of both MTT and chemotherapy agents. Moreover, we explored the functional implications of NOS2 in driving the malignant progression of HB.

**Figure 2:**
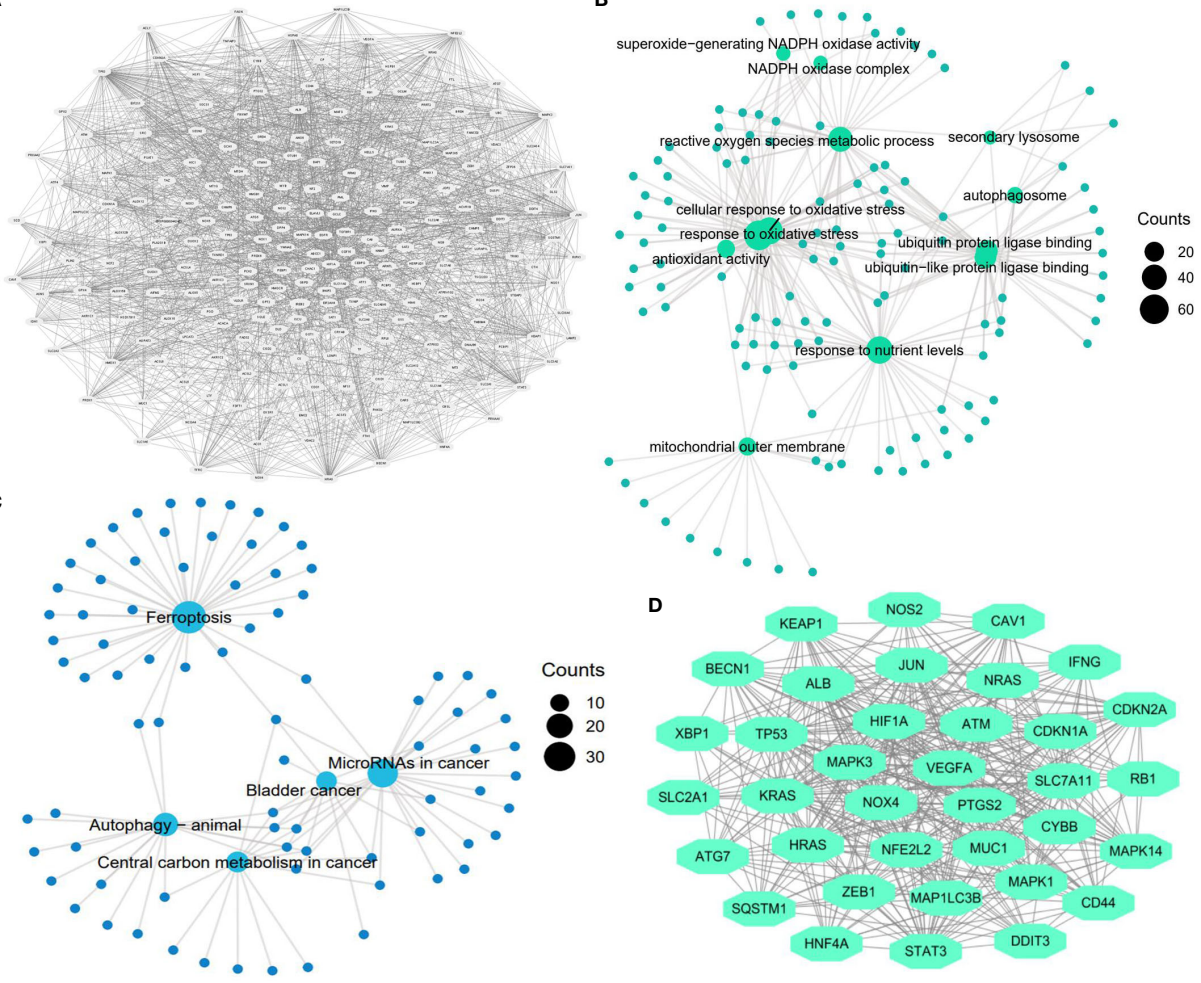
Construction of ferroptosis-related gene set. **(A)** Protein-protein interaction (PPI) network of ferroptosis genes (n=253). **(B)** GO function analysis of ferroptosis genes (n=253). **(C)** KEGG function analysis of ferroptosis genes (n=253). **(D)** The most significant gene module in the ferroptosis PPI network (n=36).

A reliable gene set is a prerequisite for finding key genes regulating cancer development (31). With the aid of the FerrDb database, we included 253 human ferroptosis genes in the FR gene set. The PPI network of these genes is exhibited in **Figure 2A**. Functional analysis revealed that these regulators were closely involved in multiple pivotal sessions of ferroptosis, such as the reactive oxygen species metabolism process, cellular response to oxidative stress, and antioxidant activity (**Figure 2B**). KEGG analysis also showed that the selected genes were highly enriched in the ferroptosis process (**Figure 2C**). These findings confirmed the reliability of the FR gene set. Through module analysis, 36 core ferroptosis genes were obtained for further analyses (**Figure 2D**).

### 3.2 Three ferroptosis genes may play critical roles in HB

**Figure 3:**
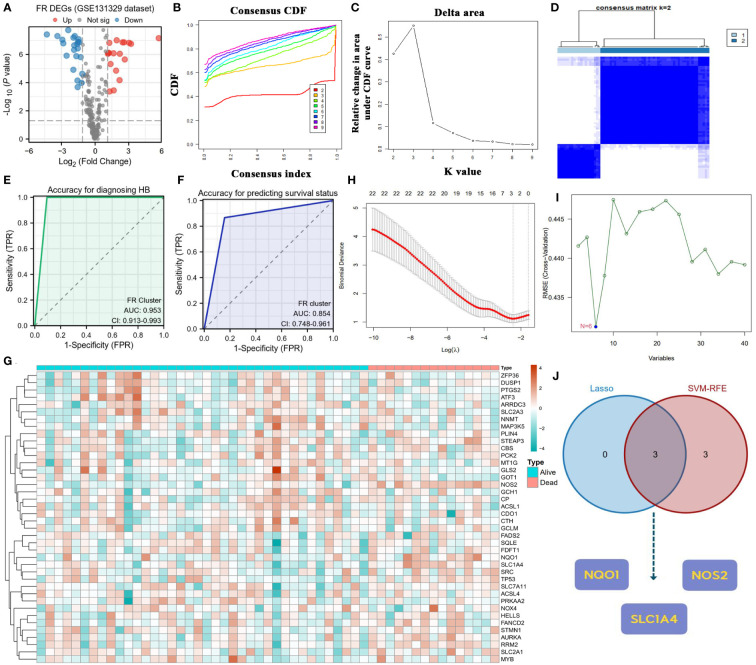
Screening core ferroptosis genes in hepatoblastoma. **(A)** Volcano plot of FR DEGs between normal and tumor samples. **(B)** CDFs in clustering analysis. **(C)** Delta area in clustering analysis. **(D)** Heatmap of the consistency matrix in clustering analysis. **(E)** Diagnostic accuracy of FR clusters in the GES131329 dataset. **(F)** Predicted accuracy of prognosis for FR clusters in the GES131329 dataset. **(G)** Expressive heatmap of ferroptosis DEGs with different survival status. **(H)** Track diagram of Lasso regression analysis. **(I)** Results of SVM-RFE analysis. **(J)** Point of intersection between Lasso regression analysis and SVM-RFE algorithm. CDF, cumulative distribution function; FR, ferroptosis-related; DEGs, differentially expressed genes; SVM-RFE, support vector machine recursive feature elimination.

Given that only the GSE131329 dataset provided the survival status of patients with HB among the three GEO datasets, we used it as the training cohort to identify the critical regulators involved in HB progression and clinical outcome. Before further bioinformatic analyses, the difference in transcriptome data of FR genes between normal and tumor samples was first determined (**Figure 3A**). Notably, 15.8% of FR genes (40/253) were differentially expressed in the HB samples. Differentially expressed genes (DEGs) in ferroptosis revealed two cluster patterns for characterizing HB (**Figures 3B–D**). The ferroptosis clusters not only demonstrated an excellent diagnostic capacity for distinguishing between tumor and normal cases (AUC=0.953, **Figure 3E**), but also had substantial potential for predicting the survival outcomes of patients with HB (AUC=0.854, **Figure 3F**).

**Figure 3G** displays the heatmap of FR DEGs based on distinct survival statuses. To identify key FR genes in HB pathogenesis, we performed Lasso regression analysis and applied the SVM-RFE algorithm using survival status as the outcome variable (**Figures 3H, I**). The genes that emerged as common outcomes from both machine-learning methods were identified as the intersection (**Figure 3J**). From this selection, NQO1, NOS2, and SLC1A4 were screened for further clinical correlation analyses.

### 3.3 The associations of NQO1, NOS2, and SLC1A4 with clinicopathological features in HB

**Figure 4:**
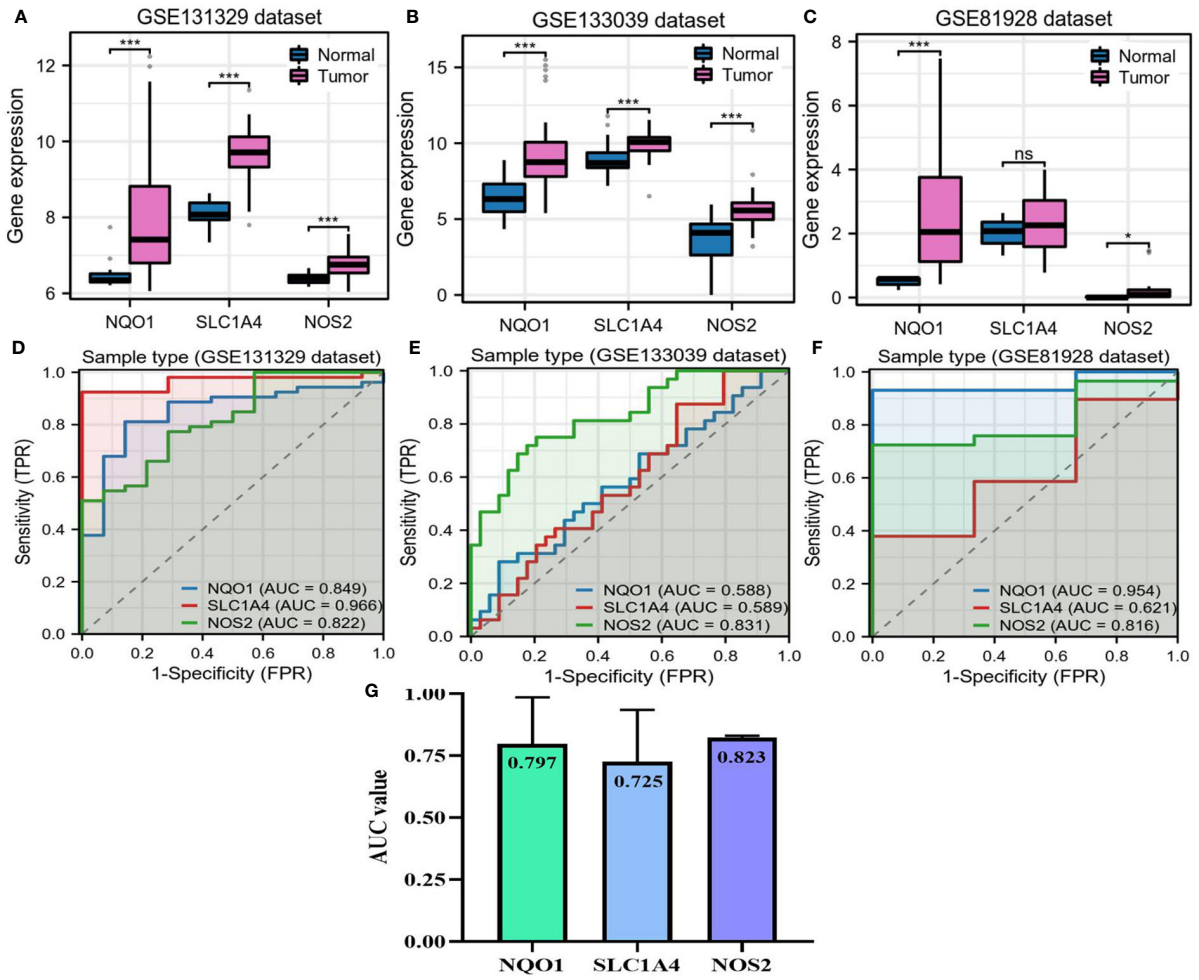
Expressions of *NQO1*, *NOS2*, and *SLC1A4* in hepatoblastoma. **(A–C)** The differences in expression of three candidate genes between HB and adjacent normal liver samples. **(D–F)** Accuracy of three candidate genes for diagnosing HB in three GEO datasets. **(G)** Mean AUC value of each candidate gene across three GEO datasets. AUC, area under the curve; HB, hepatoblastoma; TPR, true positive rate; FPR, false positive rate. NS, not significantly; *P<0.05, **P<0.01, ***P<0.001.

The basis for functional gene activity lies in their expressive differences. Hence, we confirmed the expressions of NQO1, NOS2 and SLC1A4 in HB. All three candidate genes were overexpressed in tumor samples in the GSE133039 and GSE131329 datasets (**Figures 4A, B**). However, there was no difference in SLC1A4 expression between normal and tumor samples in the GSE81928 dataset (**Figure 4C**). ROC curve analyses indicated that the expression of these genes could differentiate between normal and HB samples in most datasets (**Figures 4D-F**), with an average diagnostic accuracy of ~0.80 (**Figure 4G**). Specifically, NOS2 emerged as the best diagnostic biomarker with the highest AUC value of 0.823 and the lowest standard difference of 0.007 (**Figure 4G**).

**Figure 5:**
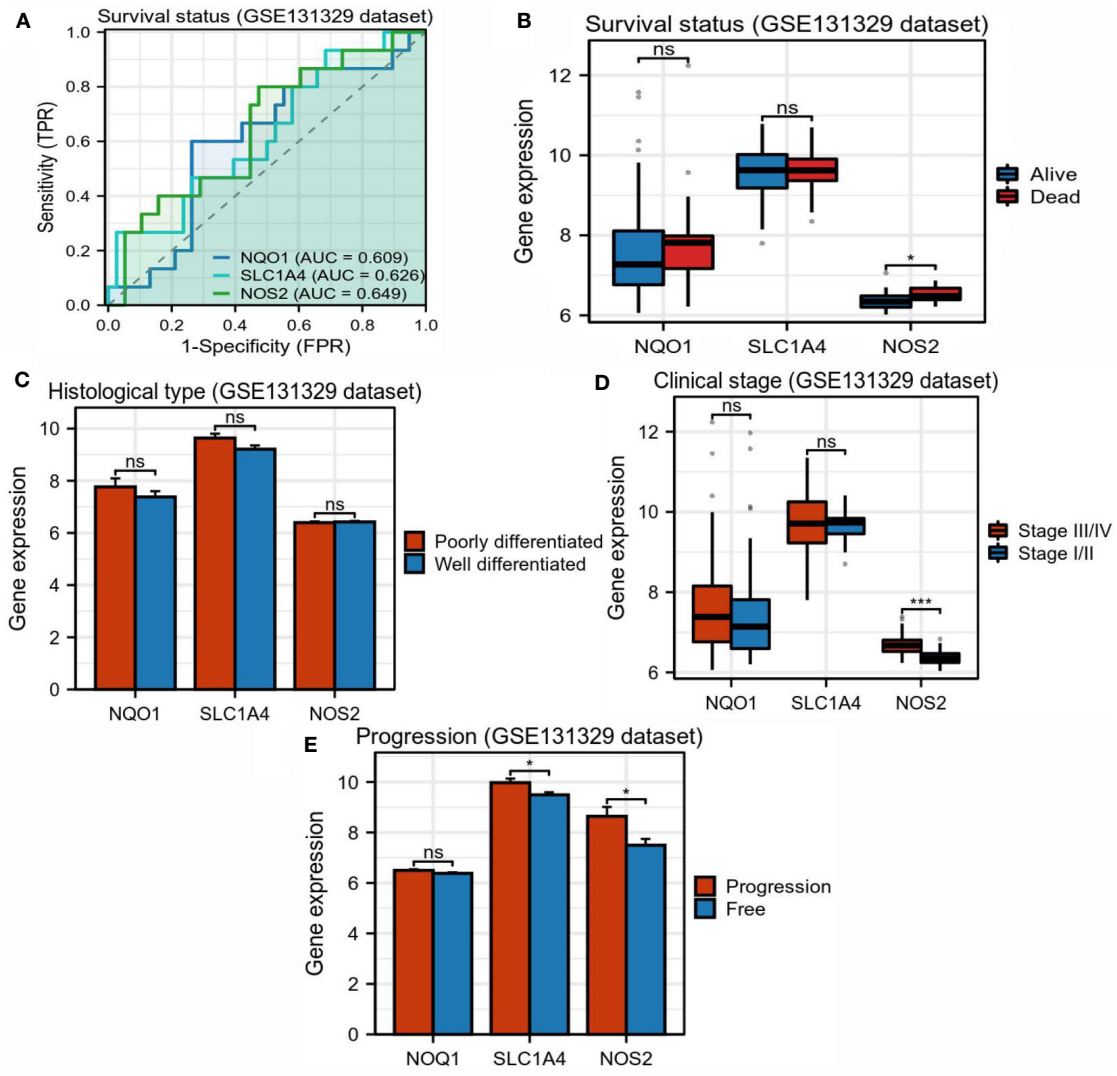
Correlations between three candidate genes and clinicopathological characteristics in hepatoblastoma. **(A)** Accuracy of NQO1, SLC1A4, and NOS2 for predicting survival outcomes of children with HB (GSE131329). **(B)** Differences in expression of three candidate genes between surviving and deceased patients with HB (GSE131329). **(C)** Differences in expression of three candidate genes between patients with poorly- and well-differentiated HB (GSE131329). **(D)** Differences in expression of three candidate genes between patients with early- and late-stage HB (GSE131329). **(E)** Differences in expression of three candidate genes between patients with metastatic and non-metastatic HB(GSE131329). HB, hepatoblastoma; NS, not significantly; *P<0.05, *P<0.01, *P<0.001.

Further, we investigated the relationships between three candidate genes and HB clinical status. In the GSE131329 dataset, NOS2 had moderate competence for predicting survival outcomes of patients with HB, which was slightly higher than NQO1 and SLC1A4 (AUC=0.649, **Figure 5A**). There were no differences in the expressions of NQO1 and SLC1A4 between different survival outcomes, whereas higher NOS2 expression was observed in surviving cases compared to deceased cases (**Figure 5B**). As for histological types, no differences in the expressions of the three genes were found between poorly- and well-differentiated patterns (**Figure 5C**). Moreover, higher NOS2 expression was concordant with metastatic events and late clinical stage in the GSE131329 dataset, while no correlations were found for NQO1 (**Figures 5D, E**). Collectively, although NQO1, NOS2, and SLC1A4 were all aberrantly expressed in HB, only NOS2 was closely associated with clinicopathological features. Therefore, we further investigated the effects of NOS2 on the tumor immune microenvironment (TIME) and metabolism in HB.

### 3.4 NOS2 expression map reveals different immune landscapes in HB

**Figure 6:**
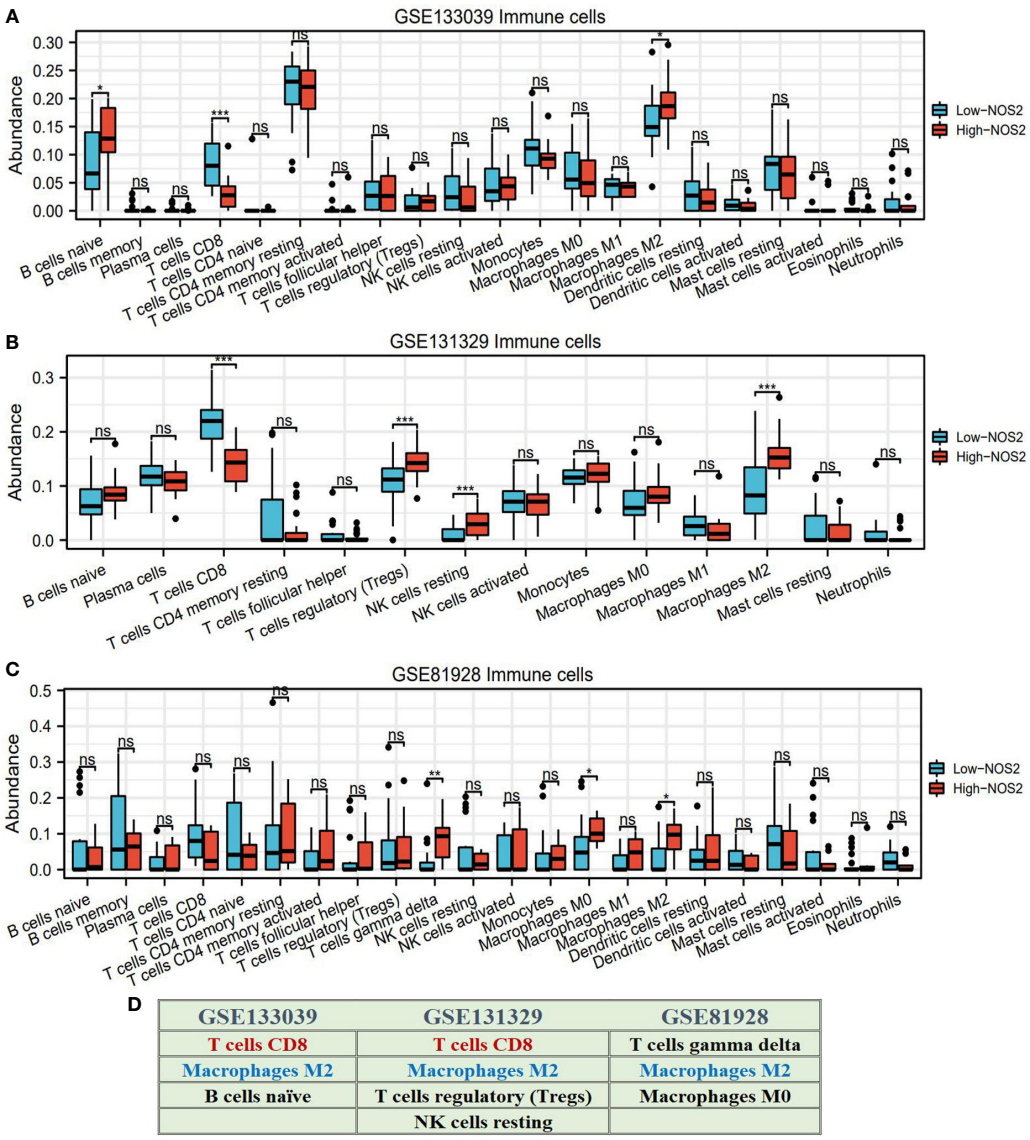
NOS2 effects on the infiltration levels of multiple immune cells based on CIBERSORT algorithm. **(A–C)** Differences in the infiltration levels of multiple immune cells between high- and low-*NOS2* expression in four GEO datasets. **(D)** Summary of NOS2 immune effect. *P<0.05, **P<0.01, ***P<0.001. NS, not statistically significant.

Through using the CIBERSORT algorithm, we found that different *NOS2* expression resulted in alteration of immune cell abundance (**Figures 6A–C**). For instance, in the GSE133039 dataset, the infiltration levels of CD8+ T cells were significantly increased in the high-NOS2 expression group compared with the low expression group, whereas that of macrophages and naïve B cells was markedly increased (**Figure 6A**). An overview of the immune analyses findings is summarized in **Figure 6D**. Notably, CD8+ T cells and macrophages were the dominant cell types affected by NOS2 expression. Given the functions of these cells in anti-tumor immune response and their variation in HB samples with high-NOS2 expression (**Table 1**), it became apparent that high-NOS2 expression indicated an unfavorable anti-tumor immune process.

**Table 1:** Effects of NOS2 on tumor immune microenvironment.

Immune cell	Variation in H group	Affected dataset	Basic function of immune cell	Final effect on anti-tumor immune
CD8+ T cells	Decreased	GSE133039 GSE131329	Eradicating tumor cells through potent cytotoxic effect	Unfavorable
Macrophage M2	Increased	GSE133039 GSE131329 GSE81928	Promoting cancer progression through secreting VEGF	Unfavorable
B I naive	Increased	GSE133039	Naïve B cells are differentiated into various kinds of B cells in TIME, such as plasma cells and memory B cells.	Uncertain
T cells regulatory	Increased	GSE131329	Tregs are involved in immune tolerance through modulating MDSCs function and suppressing excessive immune responses.	Unfavorable
NK cells resting	Increased	GSE131329	Active NK cells can suppress cancer metastases by killing circulating tumor cells. However, resting NK cells are not available for this role.	Unfavorable
T cells gamma delta	Increased	GSE81928	γδ T cells kill tumor cells in a classical MHC-independent manner.	Favorable
Macrophage M0	Increased	GSE81928	Macrophage polarization decides the final immune effects. Macrophage M1 possesses anti-tumor function, whereas M2 type has cancer-promoting abilities.	Uncertain

H group, high-NOS2 expression group; VEGF, vascular endothelial growth factor; TIME, tumor immune environment; Tregs, T cells regulatory; MDSCs, myeloid-derived suppressor cells; γδ T cells, gamma delta T cells; MHC, major histocompatibility complex.

**Figure 7:**
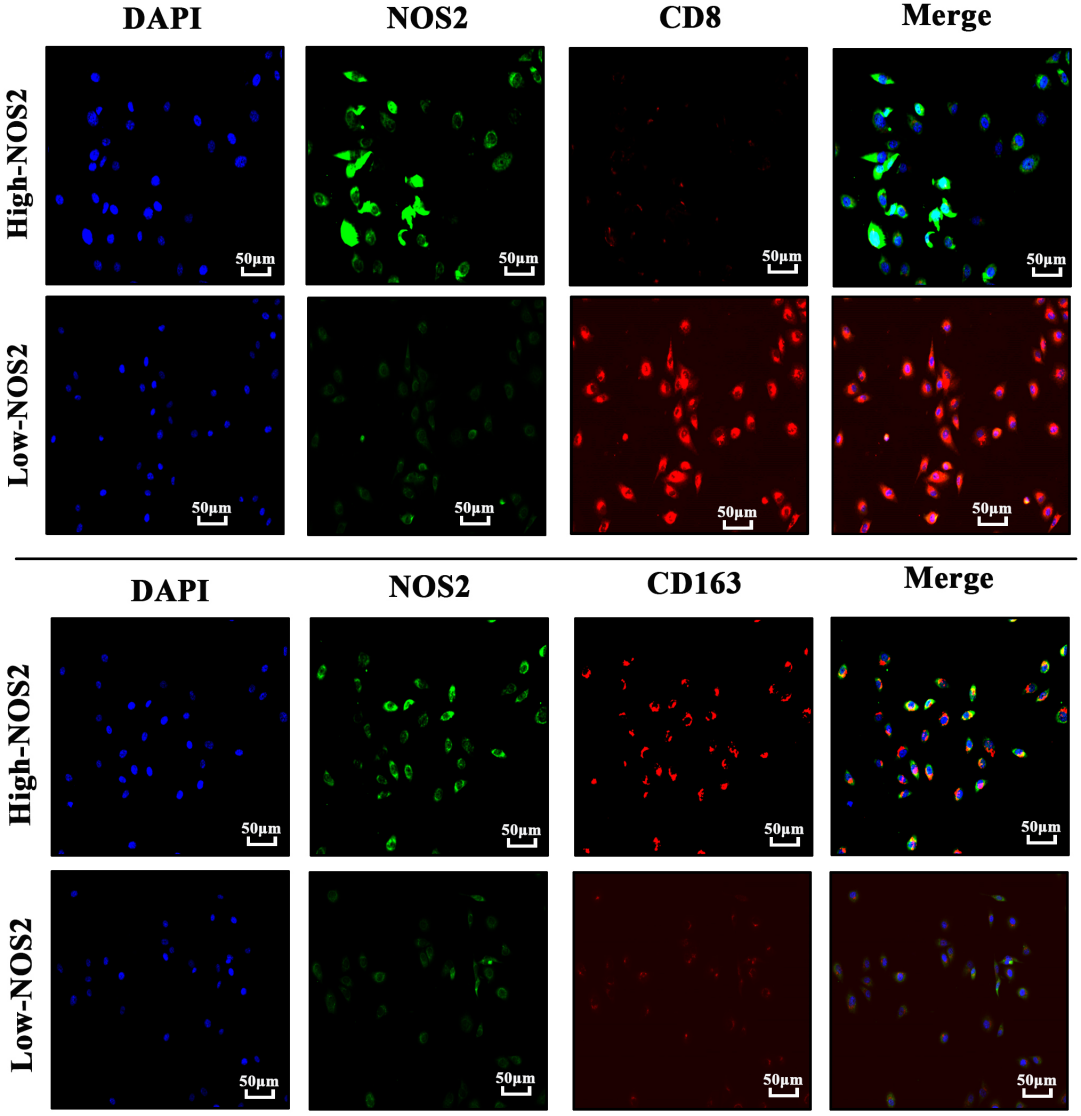
Immunofluorescence of four hepatoblastoma clinical samples. Cell nuclei are labeled by blue-fluorescence; NOS2 protein are labeled by green-fluorescence; CD8+ T cells (CD8) and macrophages (CD163) are labeled by red-fluorescence.

To verify the effects of NOS2 on the TIME in HB, we performed immunofluorescent staining on HB clinical samples with different NOS2 expression levels. As shown in **Figure 7**, the green fluorescence intensity of labelled NOS2 was consistent with NOS2 expression. However, the red fluorescence intensities of labelled CD8+T cells and macrophages exhibited vastly different levels. In samples with high-NOS2 expression, the red fluorescence of CD8+T cells was rarely visible, whereas that of macrophages filled the field of view. Inversely, in samples with low-NOS2 expression, the fluorescence intensity of CD8+ T cells was strong, whereas that of macrophages was extremely weak. These findings confirmed that NOS2 expression acted as a biomarker of immune status in HB.

### 3.5 NOS2 hardly leads to metabolic reprogramming in HB

**Figure 8:**
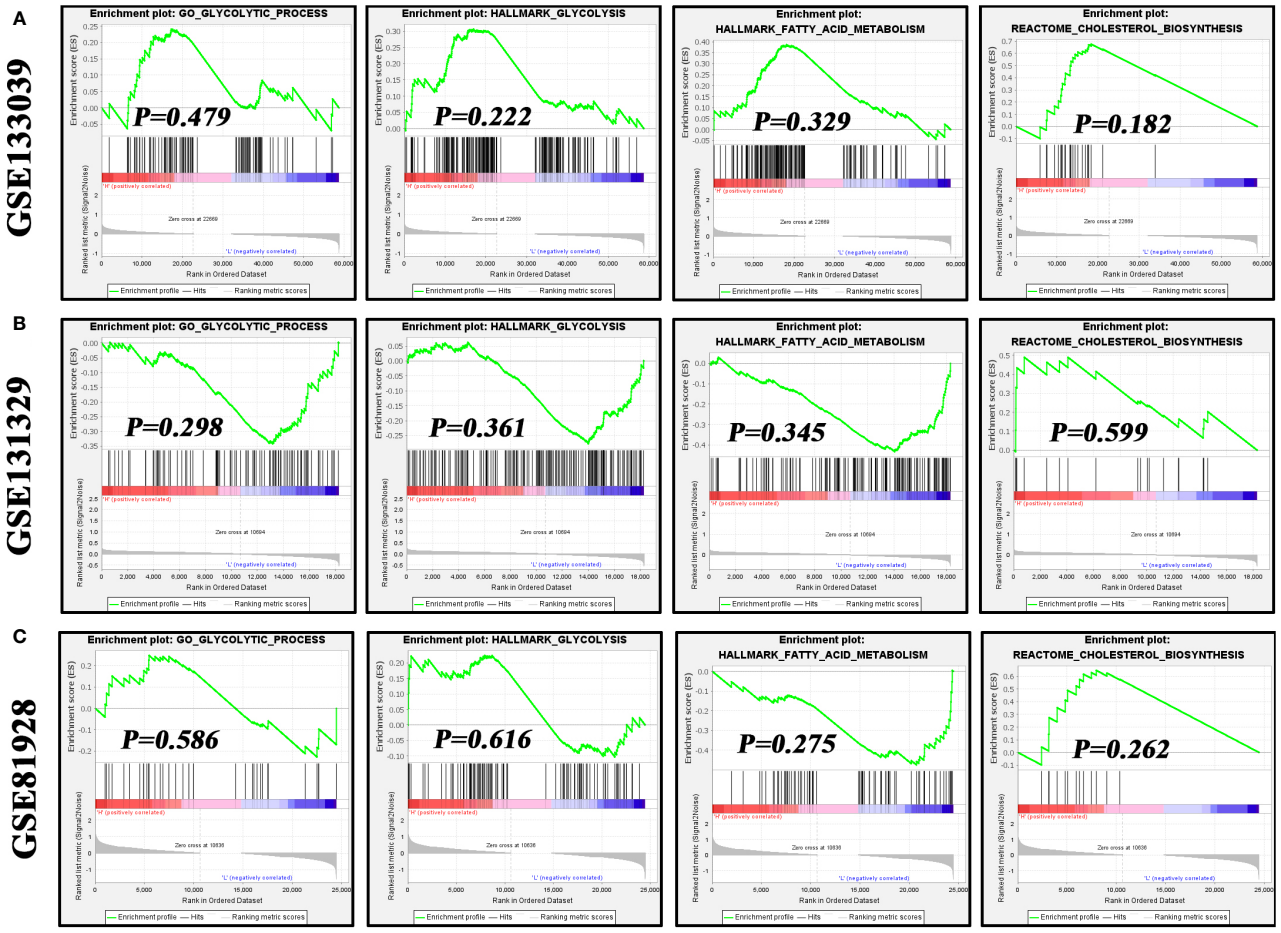
The effects of *NOS2* expression on the activities of three metabolism processes in hepatoblastoma. **(A–C)** Differences in enrichment of glycolysis, fatty acid metabolism, and cholesterol biosynthesis in the GSE133039, GSE131329, and GSE81928 datasets using GSEA. GSEA, gene set enrichment analysis.

Metabolic reprogramming, particularly characterized by enhanced glycolysis, is a critical hallmark of cancer cells (32, 33). Hence, we investigated the associations of NOS2 with three metabolic pathways, including glycolysis, fatty acid metabolism, and cholesterol biosynthesis. Interestingly, using GSEA analysis, there were no enrichment differences in these metabolic processes between samples with high- and low-NOS2 expression (**Figures 8A–C**). In light of the above, it was speculated that alterations in *NOS2* expression might not significantly impact the metabolic processes within HB metabolic.

### 3.6 NOS2 is a biomarker for predicting the efficacy of trametinib, lapatinib, and cisplatin

**Figure 9:**
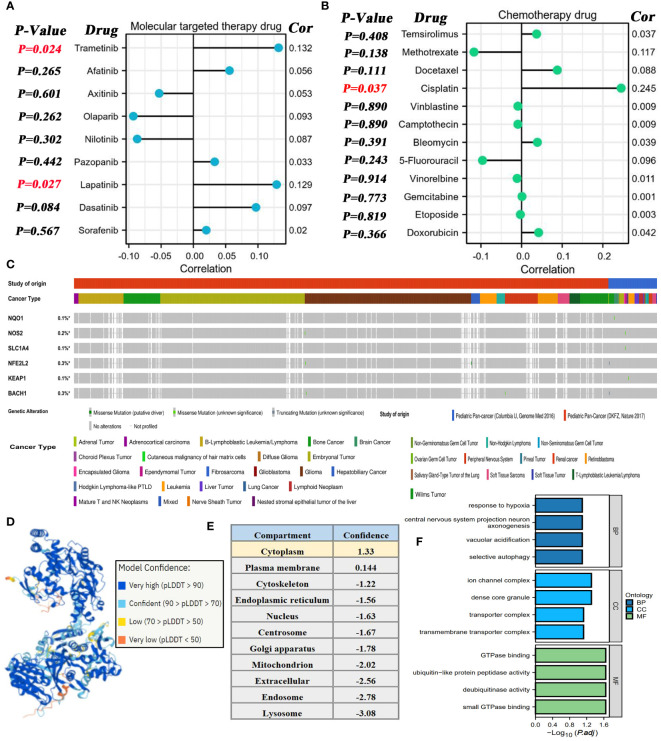
Associations of NOS2 with therapeutic efficacy of multiple drugs. **(A)** Correlations between NOS2 expression and the sensitivities (IC_50_ value) of nine commonly used MTT drugs based on the GDSC database. **(B)** Correlations between NOS2 expression and the sensitivities of 12 commonly used chemotherapy drugs based on the GDSC database. **(C)** Somatic mutation frequency and type of six pivotal genes in two pediatric pan-cancer projects based on the cBioPortal database. **(D)** Protein structure of NOX1 (AlphaFold Iabase). **(E)** Subcellular localization of NOS2. **(F)** GO analyses of NOS2-related genes.

Using the GDSC database, the potential association between NOS2 expression and the therapeutic effects of multiple drugs were explored. For MTT, NOS2 expression was not correlated with the sensitivity of most MTT drugs; however, it was positively correlated with that of trametinib and lapatinib (**Figure 9A**). Similarly, no correlations were observed between NOS2 expression and commonly used chemotherapeutic drugs, such as 5-fluorouracil, doxorubicin, and vinblastine (**Figure 9B**). Only the IC_50_ value of cisplatin was positively correlated with NOS2 expression (R=0.245, P=0.037). Therefore, high expression of NOS2 was indicative of resistance to trametinib, lapatinib, and cisplatin treatments.

Moreover, the mutational characteristics of several crucial genes (NQO1, NOS2, SLC1A4, NFE2L2, KEAP1, and BACH1) were briefly elaborated through the cBioportal database. The somatic mutations of these genes rarely appeared in pediatric cancer samples with a mutational frequency of ~0.3% (**Figure 9C**). More specifically, we also focused on their mutational status in HB samples (16 cases from the ‘Columbia U’ project and 0 cases from the ‘DKFZ’ project). As shown in **Supplementary Table 6**, only 1 HB sample was concomitant with the NFE2L2 missense mutation, whereas any mutations of the other genes were not found in all 16 HB samples.

The three-dimensional protein structure of NOS2 was obtained through the AlphaFold database (**Figure 9D**), which provided some clues for artificially synthesizing a NOS2 inhibitor. Additionally, the Hum-mPLoc online tool revealed that the cytoplasm was the main subcellular localization of NOS2 (**Figure 9E**). Functional analysis indicated that genes highly correlated with NOS2 (R≥0.5) may participate in multiple biological processes, such as ‘response to hypoxia’ and ‘GTPase binding’ (**Figure 9F**).

### 3.7 NOS2 promotes proliferative, migrative, and invasive abilities of HB cells

NOS2 acts as a biological mediator in human diseases by generating nitric oxide (NO). Numerous studies have demonstrated its roles in various types of cancers (**Table 2**). Through the above analyses, we found that NOS2 was not only closely associated with clinical features of HB, but also with its prognosis, anti-tumor immunity, and therapeutic effects. Therefore, we further ascertained the biological functions of NOS2 in HB cells.

**Table 2:** NOS2 functions in human cancers.

Cancer Type	PMID	Function	Role
Breast cancer	35740092	iNOS/NOS2 axis promotes breast cancer progression through regulating HER2, BRCA1, and BRCA2.	Oncogenic gene
Hepatocellular cancer	16273602	NOS2 facilitates tumour cell angiogenesis, invasion, and metastasis through upregulating MMP9.	Oncogenic gene
Urinary bladder cancer	26298202	NOS2 polymorphism affects the outcome of BCG treatment.	Biomarker
Lung cancer	22618808	NOS2 enhances KRAS-induced lung carcinogenesis.	Oncogenic gene
Colon cancer	34719858	Knockdown of NOS2 splicing variant S3 induces cell autophagy.	Oncogenic gene

**Figure 10:**
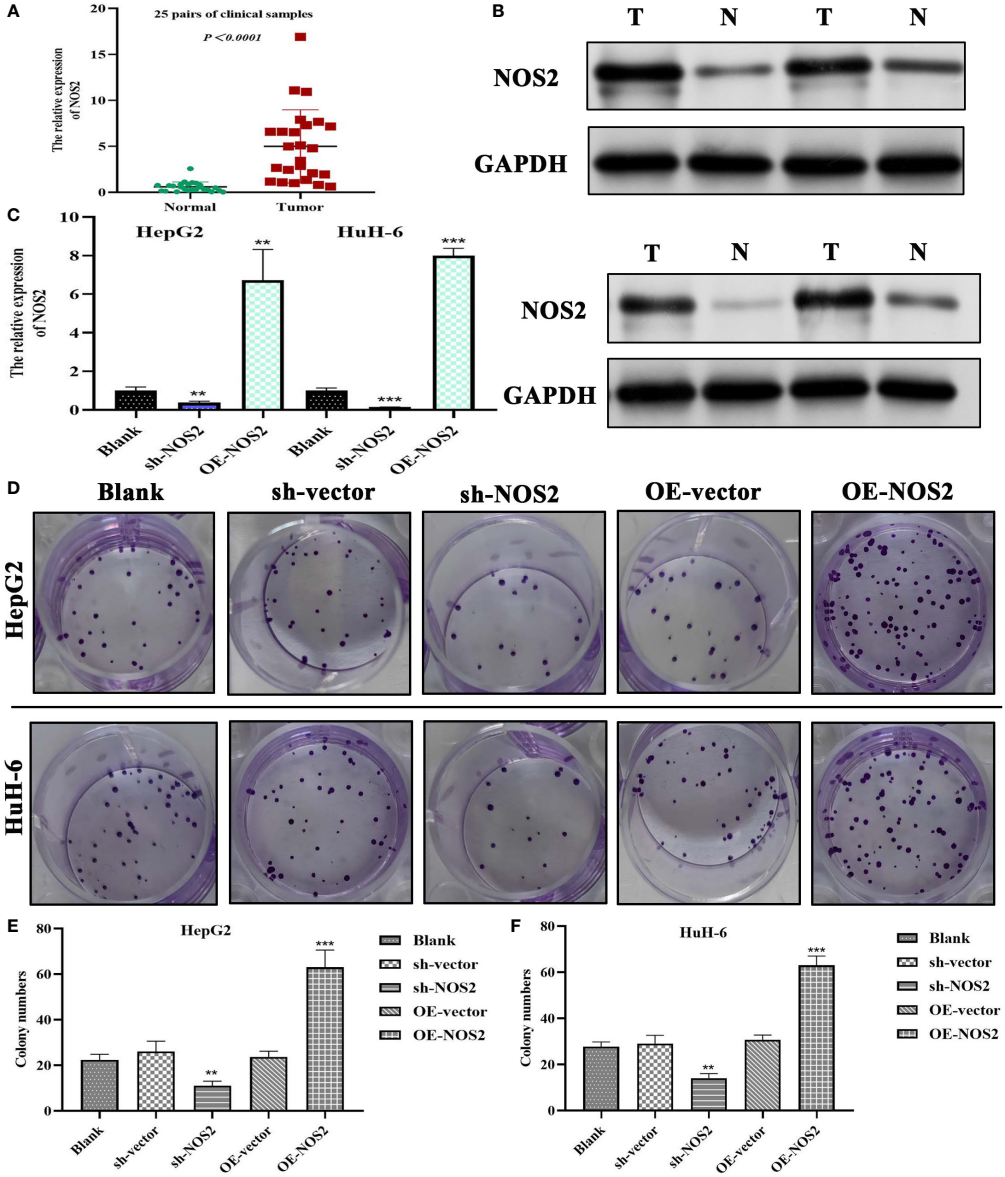
NOS2 promotes the proliferation of hepatoblastoma cells. **(A)** PCR tests on 25 pairs of clinical samples. **(B)** Western blot detection on four pairs of clinical samples. **(C)** Tests of transfection efficiency of sh-NOS2 and OE-NOS2 in HepG2 and HuH-6 cells. **(D)** Results of colony formation assays in two hepatoblastoma cells. **(E, F)** Colony numbers of five experimental groups in HepG2 and HuH-6 cells. sh, short hairpin; OE, over-expression; sh-vector and OE-vector are negative control groups. **P<0.01, ***P<0.001.

Through RT-qPCR (n=25) and Western blot (n=4) analysis on clinical samples, we successfully verified the upregulation of NOS2 in HB (**Figures 10A, B**). Further experiments involving transfection with specific plasmids (i.e., sh-NOS2 and OE-NOS2), effectively altered *NOS2* expression in HepG2 and HuH-6 cells (**Figure 10C**). Colony formation assays showed that *NOS2* knockdown significantly inhibited cell clone formation, whereas *NOS2* overexpression markedly promoted this process (**Figure 10D**). Similar results were also found in the quantitative analyses (**Figures 10E, F**). Hence, NOS2 could enhance the proliferative abilities of HepG2 and HuH-6 cells.

**Figure 11:**
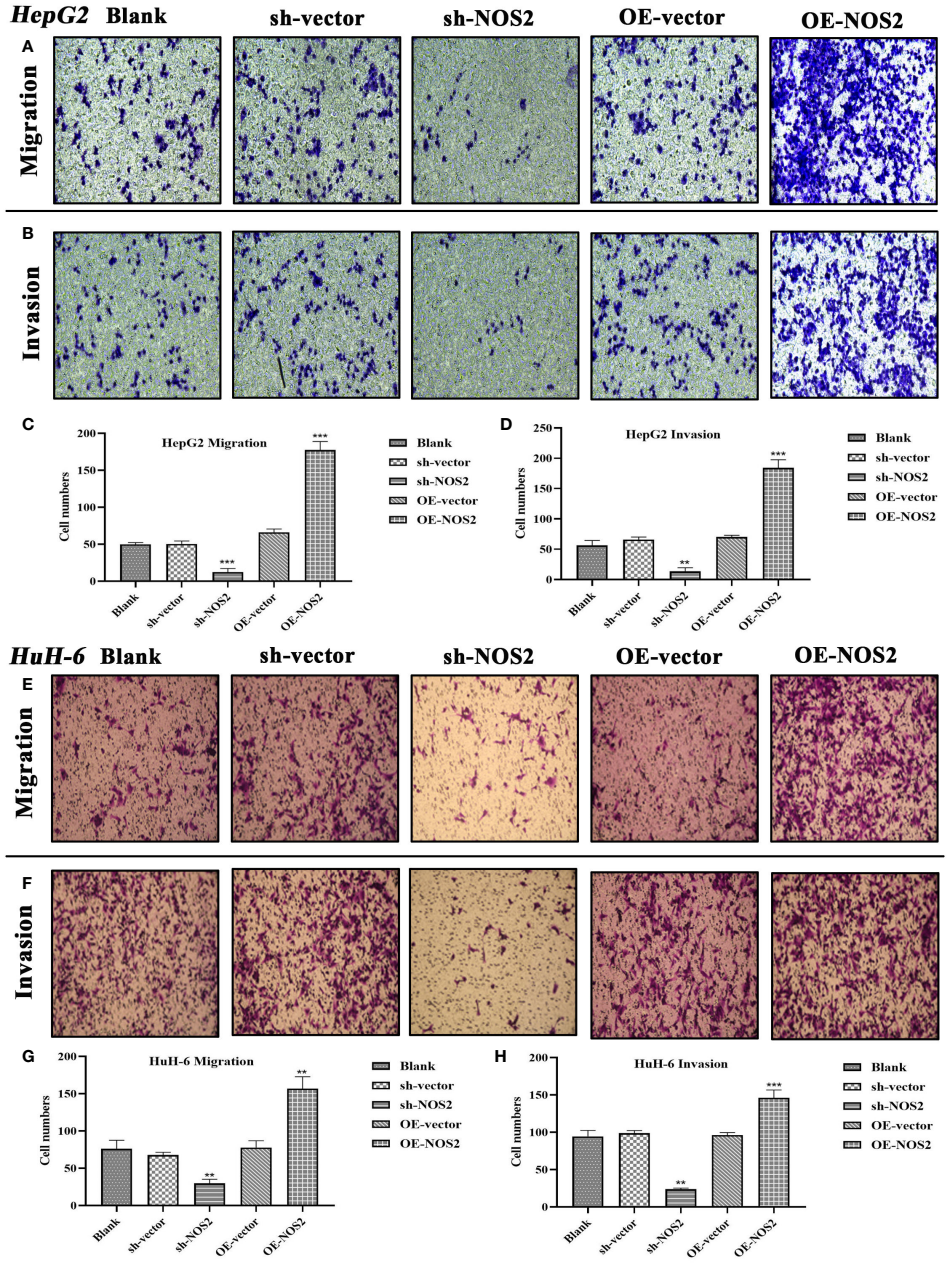
NOS2 enhances the migrative and invasive abilities of hepatoblastoma cells. **(A, B)** Transwell assays are applied to assess the effects of NOS2 on migration and invasion of HepG2 cells. **(C, D)** Quantitative statistics for Transwell migration and invasion assays in HepG2 cells. **(E, F)** Transwell assays are applied to assess the effects of NOS2 on migration and invasion of HuH-6 cells. **(G, H)** Quantitative statistics for Transwell migration and invasion assays in HuH-6 cells. **P<0.01, ***P<0.001.

Next, we verified the effects of NOS2 on the migration and invasion of HB cells through Transwell assays. In HepG2 cells, the migrative and invasive cells were significantly increased in the overexpression group (OE-NOS2) compared to the control group (**Figures 11A, B**). However, migration and invasion were markedly decreased in the NOS-silenced group (sh-NOS2) (**Figures 11A, B**). The quantitative analyses, based on cell counts, were also consistent with the above findings (**Figures 11C, D**). Similarly, in HuH-6 cells, NOS2 overexpression increased migration and invasion of cells, whereas *NOS2* deletion exerted an inhibitory effect (**Figures 11E–H**). Collectively, NOS2 was shown to possess cancer-promoting abilities within HB cells, significantly influencing their migration and invasion capacities.

### 3.8 Potential upstream regulatory mechanisms of NOS2

**Figure 12:**
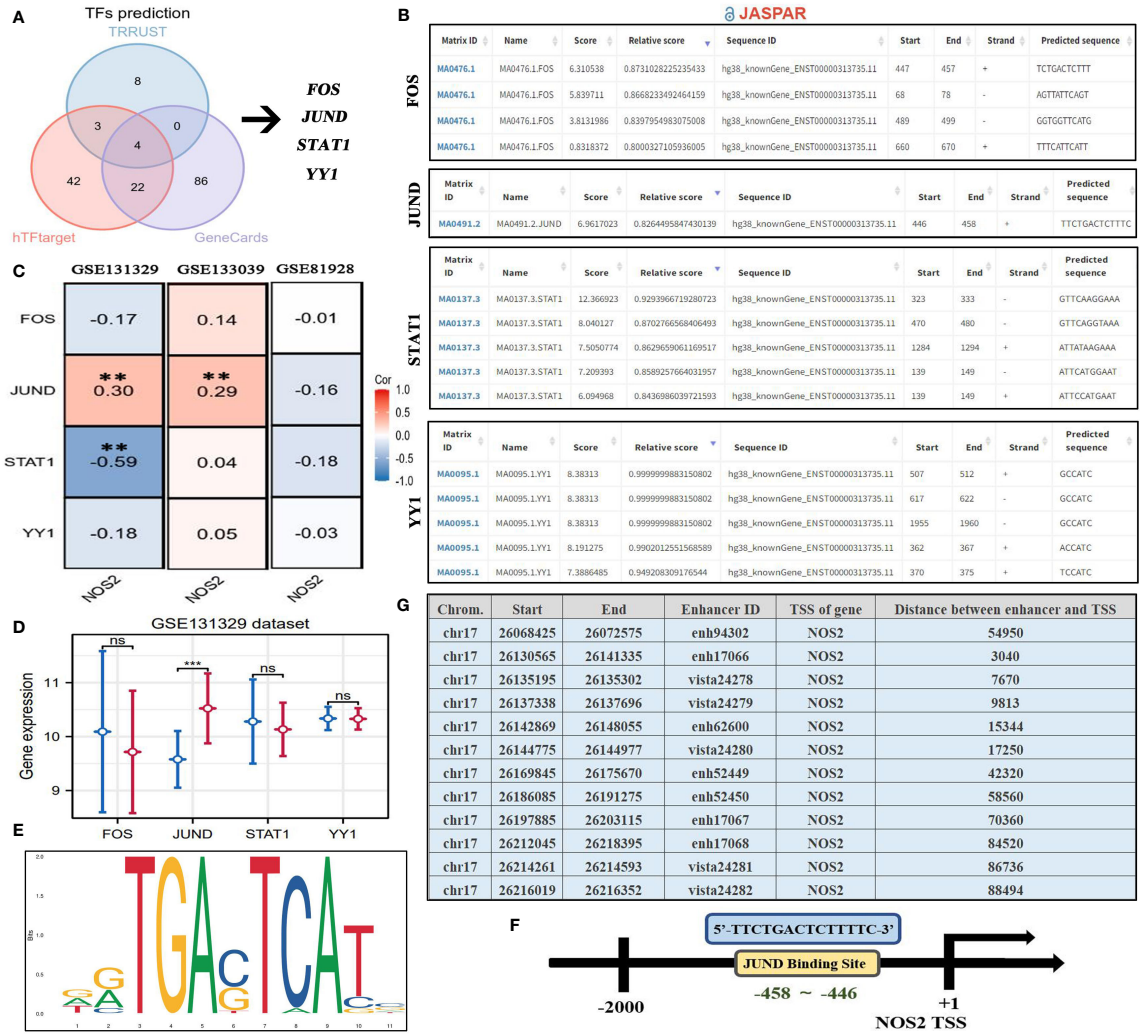
Upstream regulatory mechanism of NOS2. **(A)** Four TFs are predicted as the potential upstream regulators of NOS2 through TRRUST, hTFtarget, and GeneCards databases. **(B)** Potential binding sites between four TFs and NOS2 predicted by the JASPAR database. **(C)** Expressive correlations between NOS2 and four TFs among three GEO datasets. **(D)** The differences in levels of four TFs between deceased and surviving cases in the GSE131329 dataset. **(E)** Motif sequence of JUND obtained from the JASPAR database. **(F)** JUND transcriptional regulate NOS2 through binding its promoter (5’-TTCTGACTCTTTT-3’). **(G)** Potential enhancers regulating NOS2 (EnhancerDB database). TFs, transcription factors; TSS, transcription start site; **P<0.01. ***P<0.001; NS, not statistically significant.

Using the TRRUST, hTFtarget, and GeneCards databases, we predicted upstream TFs of NOS2.By merging the results using a Venn diagram, we identified serval candidate TFs, including FOS, JUND, STAT1, and YY1 (**Figure 12A**). The JASPAR database was used to quantify the probabilities of these TFs binding to the NOS2 promoter via predictive scores (**Figure 12B**). Among these, STAT1 had the highest predictive score of 12.366, indicating that STAT1 may play a role in transcriptional regulation of NOS2. Curiously, only JUND had a positive expression correlation with NOS2, while STAT1 demonstrated a negative correlation (**Figure 12C**). There were no significant differences in the expressions of FOS, STAT1, and YY1 between patients with HB that had deceased or surviving outcomes, except for JUND (**Figure 12D**). Therefore, despite STAT1 having the highest predictive score, JUND seemed to be more likely to regulate NOS2. The motif sequence of JUND’s potential binding to NOS2 is presented in **Figure 12E**. The binding site was identified as 5’-TTCTGACTCTTTT-3’, located between the 458^th^ base to the 446^th^ base positions upstream of the NOS2 transcription start site (TSS) (**Figure 12F**).

Of note, the JASPAR database can only infer the binding possibility between TFs and the promoter regions. It fails to predict interactions between TFs and enhancer regions. Therefore, the above analyses could not exhibit a comprehensive view of the upstream regulatory landscape of NOS2. Thus, to address the above shortfall, we preliminarily investigated the enhancers that regulated NOS2 through the EnhancerDB database (**Figure 12G**). The results revealed that a total of 12 enhancers may be involved in NOS2 regulation. The distance between enhancers and the NOS2 TSS ranged from 3,040 kb to 88,494 kb. This approach offers a broader perspective on the potential regulatory elements influencing NOS2, capturing both promoter-based and enhancer-based interactions.

### 3.9 Comparison between NOS2 and other existing biomarkers in HB

To date, some studies have developed new biomarkers for HB clinical assessments. In comparison with these investigations, NOS2 offers several distinct advantages (**Table 3**). First, NOS2 exhibits the excellent diagnostic performance. Recently, Liu et al. and Jiao et al. have reported that exosomal miR-21 and miR-34 were upregulated in HB samples, thereby acting as potential diagnostic biomarkers (34, 35). NOS2 diagnostic accuracy is comparable to that of above biomarkers (0.823 versus 0.861 and 0.837). Second, NOS2 comprehensively demonstrates the clinical significance of biomarkers. Previous studies primarily focused on establishing the relationships between biomarkers and HB prognosis. For instance, Zhao et al. exclusively confirm the expression pattern of apolipoprotein A-I in HB without performing any clinical correlation analysis (36). By contrast, our study not only investigated the prognostic value of NOS2, but also elaborated its associations with immune response, metabolism, and therapeutic effects. Third, NOS2 provides insights into the biological functions of biomarkers. In the present study, we employed a series of *in vitro* experiments to ascertain the effects of NOS2 on HB proliferation, migration, and invasion. These findings provide new clues for the identification of novel therapeutic targets. In summation, despite the existence of identified HB biomarkers, NOS2 significantly enriches our understanding of HB clinical assessments, offering valuable information with its distinct advantages.

**Table 3:** Comparison between NOS2 and other hepatoblastoma biomarkers.

Biomarkers	PMID	Diagnostic performance	Clinical correlation	Biofunction
SERPINE2	32189106	NA	Prognosis	Yes
miR-21	27601233	0.861	Prognosis	NA
Circ 0000594	31646606	NA	Malignant progression	Yes
miRNA-34	28277300	0.837	Prognosis	NA
Apolipoprotein A-1	26171005	NA	Expressive trend	NA
NOS2	NA	0.823	Malignant progression, prognosis, immune effect, metabolism, and therapeutic correlation	Yes

NA, not applicable.

## 4 Discussion

As the most common liver tumor affecting children, HB not only poses significant health risks to these young patients but also imposes a substantial psychological burden on their parents (37). Due to its easy metastasis and high malignancy, up to 10% of children may require hepatic transplantation to achieve clinical cure (38). Although a comprehensive treatment consisting of surgical resection and chemotherapy has contributed to a notable improvement in the 5-year OS rate, reaching ~75%, there remains a concerning ~20% subset of children who are ineligble for surgery and consequently experience a 5-year OS rate of just 55% (38). Therefore, developing and refining the current therapeutic strategy is of paramount importance. Herein, our study found that NOS2 acted as a pivotal biomarker capable of reflecting disease status, eliciting immune response, as well as predicting therapeutic efficacy and malignant progression in HB. This discovery offers profound insights that greatly inspire and impact the clinical assessment and treatment of HB. By providing a multifaceted perspective on this disease, NOS2 has the potential to revolutionize our approach to HB management, aiming to enhance outcomes for affected children and their families.

A previous study reported that mRNA expression of iNOS in HCC cells was almost undetectable (39). However, our findings revealed that NOS2 was significantly upregulated in HB. There are two possible reasons for this discrepancy. First, the differences in cell origin and pathological features play a crucial role. HB is an embryonal tumor, arising from a hepatocyte precursor (40). By contrast, HCC originates from hepatocytes or intrahepatic bile duct epithelial cells. Despite some morphologic overlap between HB and HCC, there are distinctive molecular and immunohistochemistry differences that contribute to their differentiation (41). For instance, nuclear expression of β-catenin is strongly positive in HB, but commonly negative in HCC (42). Besides, up to 90% of HB cases are associated with CTNNB1 mutation, while suchs mutations are sporadic in HCC cases (42). These findings underscore the distinct genetic, molecular, and cellular bases of HB and HCC. It is worth nothing that the HepG2 cell line commonly used in HB research is derived from the cancer tissue of a 15-year-old Caucasian male with a well-differentiated HCC. Clearly, the HepG2 cell line might not fully simulate the disease status of HB. Second, gene expressions in cell lines may not always replicate those in clinical samples. Genes are regulated by multiple biological elements within the human body. In contrast, cell line experiments typically focus on isolated factors, such as hypoxia and pharmacological interventions. For instance, PDXK is significantly upregulated in clinical samples of HCC and its high expression confers poor survival outcomes (43). However, PDXK is not overexpressed in all HCC cell lines; it exhibits weak expressions in certain cell lines like CCC-HEL-1 and BEL-7402 (43). Taken together, despite almost undetectable expression of iNOS in some HCC cell lines (39), our clinical detection still confirmed the overexpression of NOS2 in HB.

The TIME plays a pivotal role in various biological processes related to cancer, including anti-tumor immune responses and the efficacy of immunotherapy (44). Using the CIBERSORT algorithm and immunofluorescent detection, we found that high NOS2 expression was concomitant with decreased infiltration levels of CD8+ T cells and increased the levels of macrophages. Hence, increased NOS2 expression is indicative of an increased risk for an unfavorable anti-tumor immune response. Available evidence has indicated that intracellular NO regulated by NOS2 can induce polarizing macrophages from the M1 to M2 phenotypes by down-regulating interleukin‐10 levels (45). M2-type macrophages, often referred to as alternatively activated macrophages, promote cancer due to their heightened expression of molecules like vascular endothelial growth factor (46). These observations offer possible reasons for the potential adverse effects of NOS2 on the anti-tumor immune response.

Glycolysis is an essential component of metabolic reprogramming in cancers. Since it can fulfill the requirements for strong proliferation, maintain a high ratio of ATP/ADP for stimulating cell proliferation, and induce therapeutic resistance through increasing lactic acid level, glycolysis commonly remains highly metabolically active in cancers (33, 47). Interestingly, glycolysis enrichments were not affected by NOS2 expression, which contradicted our initial assumptions. Douguet et al. have found that NOS2 could improve glycolysis of peripheral γδ T cells, suggesting a potential connection between NOS2 and glycolysis (48). Given this discrepancy, it becomes imperative to delve further into this topic to unravel the potential interplay between NOS2 and glycolysis.

Cisplatin-based chemotherapy remains the mainstream therapeutic approach for managing HB (49). Although the overall objective response rate is up to ~70%, a substantial proportion of children (54%–80%) develop chemotherapy resistance after four to five cycles of treatments (50). In the present study, we found that NOS2 expression was positively correlated with the IC_50_ value of cisplatin, which suggested that patients with high-NOS2 expression might exhibit an unfavorable therapeutic response to cisplatin. Therefore, targeting NOS2 may relieve cisplatin resistance in HB. This observation could be attributed to the phenomenon of NO-releasing mediated by cellular thiols (51). Cellular thiols, predominantly represented by glutathione, can significantly reduce cisplatin-induced DNA damage, thereby mediating cisplatin resistance (52). Interestingly, increased intracellular thiols are conducive to NO generation, which is catalyzed by upregulated NOS2 (53). As a result, NOS2 emerges as a potential biomarker for predicting the efficacy of cisplatin therapy.

NOS2 is a nitric oxide synthase which is mainly expressed in the liver and can be induced by a combination of lipopolysaccharide and certain cytokines. A host of evidence highlights that NOS2 is highly expressed in most human cancers, and contributes to cancer stemness, metastasis, chemoresistance, and immunosuppression (54). For instance, miR-939-5p suppresses triple-negative breast cancer progression by inhibiting NOS2 expression (55). In pancreatic carcinoma, NOS2 enhances tumor aggressiveness by activating kynurenine metabolic signaling (56). In the context of HB, our study confirmed the oncogenic functions of NOS2 for the first time, providing a new potential therapeutic strategy for HB treatment. Notably, recent studies have demonstrated the possibility of targeting NOS2 in cancer therapy. For example, NOS2 inhibitors can assist photodynamic therapy to eradicate cancer cells in animal models of prostate cancer (57) and breast cancer (58). Likewise, our study revealed NOS2 as a promising candidate for expanding the arsenal of treatment options against HB and other cancers.

Regardless, the findings of our study is not without notable limitations. First, the clinical significance of NOS2 remains to be tested in a real clinical cohort of patients with HB. Second, AFP is a critical indicator for HB prognosis and recurrence; however, there was no available public data to investigate the relationship between NOS2 and AFP. Third, although NOS2 was associated with the drug sensitivity of cisplatin and doxorubicin, further experimental validation is required. Lastly, the detailed regulatory mechanisms of NOS2 are still elusive. Collectively, further studies need to be conducted to fully realize the potential applications of NOS2 in HB assessment and treatment.

## 5 Conclusions

The discovery of NOS2 as a potential ferroptotic biomarker in HB significantly expands our understanding of cancer onset and progression, and has important implications for clinical assessment and treatment strategies. Our study also showed that high-*NOS2* expression was associated with poor prognosis in patients with HB, unfavorable anti-tumor immune response, and resistance to cisplatin therapy. These findings enhance our understanding of the clinical landscape of HB, thereby propelling personalized medicine. It is reasonable to anticipate that NOS2 could serve as a formidable tool in the battle against HB.

## Data availability statement

The datasets presented in this study can be found in online repositories. The names of the repository/repositories and accession number(s) can be found in the article/**Supplementary Material**.

## Ethics statement

Ethical approval was not required for the studies on humans in accordance with the local legislation and institutional requirements because only commercially available established cell lines were used.

## Author contributions

YG and BY conceived and designed the study. LZ, B-CR, FW, and YL analyzed and interpreted the data. LZ, B-CR, FW, and YL wrote the manuscript. LZ, B-CR, FW, YL, and BY conducted the *in vitro* experiments. LZ, B-CR, YG, and BY revised the manuscript. All authors contributed to the article and approved the submitted version.

## Supplementary material

Supplementary Table 6 Detailed mutation information of six pivotal genes in hepatoblastoma.
