# Supplementary material for: Ferroptosis regulator NOS2 is closely associated with the prognosis and cell malignant behaviors of hepatoblastoma: a bioinformatic and in vitro study
Source: Front Oncol. 2023 Sep 19;13:1228199. doi: 10.3389/fonc.2023.1228199 (PMC10546316; doi:10.3389/fonc.2023.1228199)
Supplement: Supplementary file 1 [file Table_1.docx]

Supplementary table 1. Information and use of three datasets in this study

| Dataset | PMID | Platforms | Sample size  (T/N) | Use |
| --- | --- | --- | --- | --- |
| GSE133039 | 32240714 | GPL16791 | 34/32 | Diagnosis, expression, immune effect, and metabolism analyses |
| GSE131329 | NA | GPL6244 | 53/14 | Diagnosis, expression, clinical correlation analyses |
| GSE81928 | 30271949 | GPL16791 | 29/3 | Diagnosis and expression analyses |

NA, not available.
