# Supplementary material for: Ferroptosis regulator NOS2 is closely associated with the prognosis and cell malignant behaviors of hepatoblastoma: a bioinformatic and in vitro study
Source: Front Oncol. 2023 Sep 19;13:1228199. doi: 10.3389/fonc.2023.1228199 (PMC10546316; doi:10.3389/fonc.2023.1228199)
Supplement: Supplementary file 2 [file Table_2.docx]

Supplementary table 2. Clinical characteristics of GSE133039 and GSE131329 datasets

| Items | GSE133039 | GSE131329 |
| --- | --- | --- |
| Sample size | 66 | 67 |
| Tumor | 34 (79.1%) | 53 (79.1%) |
| Normal | 32 (79.1%) | 14 (20.9%) |
| Age (Mean value) | 17.5 months | 27.3 months |
| Gender |  |  |
| Male | 40 (59.7%) | 34 (50.7%) |
| Female | 26 (40.3%) | 33 (49.3%) |
| Survival outcomes | NA |  |
| Alive | **/** | 38 (56.7%) |
| Dead | **/** | 15 (22.4%) |
| Unknown | **/** | 14 (20.9%) |
| Histologic type |  |  |
| Well differentiated | **/** | 44 (65.7%) |
| Poorly differentiated | **/** | 21 (31.3%) |
| Unknown | **/** | 2 (3%) |
| Clinical stage | NA |  |
| Stage I | **/** | 9 (13.4%) |
| Stage II | **/** | 15 (22.4%) |
| Stage III | **/** | 18 (26.9%) |
| Stage IV | **/** | 11 (16.4%) |
| Unknown | **/** | 14 (20.9%) |
| Distant metastasis | NA |  |
| Yes | **/** | 14 (20.9%) |
| No | **/** | 39 (58.2%) |
| Unknown | **/** | 14 (20.9%) |

NA, not available.
