# Supplementary material for: Ferroptosis regulator NOS2 is closely associated with the prognosis and cell malignant behaviors of hepatoblastoma: a bioinformatic and in vitro study
Source: Front Oncol. 2023 Sep 19;13:1228199. doi: 10.3389/fonc.2023.1228199 (PMC10546316; doi:10.3389/fonc.2023.1228199)
Supplement: Supplementary file 3 [file Table_3.docx]

Supplementary table 3. Clinical characteristics of GSE81928 dataset

| Items | GSE81928 |
| --- | --- |
| Sample size | 32 |
| Tumor | 29 (90.6%) |
| Normal | 3 (9.4%) |
| Gender |  |
| Male | 20 (9.4%) |
| Female | 3 (9.4%) |
| Unknown | 9 (9.4%) |
| Age (Mean value) | 2.25 year |
| Clinical stage | NA |
| Stage I | **/** |
| Stage II | **/** |
| Stage III | **/** |
| Stage IV | **/** |
| Unknown | **/** |

NA, not available.
