# Supplementary material for: Ferroptosis regulator NOS2 is closely associated with the prognosis and cell malignant behaviors of hepatoblastoma: a bioinformatic and in vitro study
Source: Front Oncol. 2023 Sep 19;13:1228199. doi: 10.3389/fonc.2023.1228199 (PMC10546316; doi:10.3389/fonc.2023.1228199)
Supplement: Supplementary file 4 [file Table_4.docx]

Supplementary table 4. The detailed description of the gene sets used in metabolic analyses.

| Names | Gene counts | Description |
| --- | --- | --- |
| GO glycolytic process | 106 | Fermentation that includes the anaerobic conversion of glucose to pyruvate via the glycolytic pathway. |
| Hallmark Glycolysis | 200 | Genes encoding proteins involved in glycolysis and gluconeogenesis. |
| Hallmark Fatty acid Metabolism | 158 | Genes encoding proteins involved in metabolism of fatty acids. |
| Reactome Cholesterol Biosynthesis | 27 | Cholesterol biosynthesis. |
