# Supplementary material for: Ferroptosis regulator NOS2 is closely associated with the prognosis and cell malignant behaviors of hepatoblastoma: a bioinformatic and in vitro study
Source: Front Oncol. 2023 Sep 19;13:1228199. doi: 10.3389/fonc.2023.1228199 (PMC10546316; doi:10.3389/fonc.2023.1228199)
Supplement: Supplementary file 5 [file Table_5.docx]

Supplementary Table 5. The primer lists

| Gene | Primer | Sequence (5' -> 3') |
| --- | --- | --- |
| NOS2 | Forward | 5′-ATCTTGGAGCGAGTTGTGGATTGTC-3′ |
|  | Reverse | 5′-CTGGGAGGAGCTGATGGAGTAGTAG-3 |
| GAPDH | Forward | 5'‐GTCGCCAGCCGAGCCACATC‐3 |
|  | Reverse | 5'‐CCAGGCGCCCAATACGACCA‐3' |
